# Are Children's Hospitals Unique in the Community Benefits They Provide? Exploring Decisions to Prioritize Community Health Needs Among U.S. Children's and General Hospitals

**DOI:** 10.3389/fpubh.2020.00047

**Published:** 2020-02-27

**Authors:** Berkeley Franz, Cory E. Cronin

**Affiliations:** ^1^Heritage College of Osteopathic Medicine, Ohio University, Athens, OH, United States; ^2^Ohio University, Athens, OH, United States

**Keywords:** hospitals, children, pediatrics, community benefit, health policy

## Abstract

The Affordable Care Act expanded community benefit requirements for nonprofit hospitals, which now must demonstrate that they take into account the needs of their surrounding community in deciding where to make community benefit investments. No study to date has assessed the Community Health Needs Assessments (CHNAs) of a large sample of nonprofit hospitals to understand how hospitals determine the priority health needs that they include for their community or how prioritized health needs differ between general and children's hospitals. We analyzed the CHNAs of a 20% random sample of general hospitals in the United States as well as all children's hospitals. After identifying the five most common needs across all hospitals—mental health, substance misuse, social needs, chronic illness, and access to care—we used descriptive statistics and multivariate logistic regression to determine which hospitals were most likely to prioritize each of these five needs in their CHNA and the organizational, county, and regional factors associated with prioritizing a need. We found that children's hospitals were more likely than general hospitals to prioritize each of these five needs in their CHNA and that related county-level health indicators were significantly associated with hospitals prioritizing social needs and substance misuse as top needs in their CHNAs. County-level demographic variation, such as the percentage of white residents, and regional location were significantly related to whether hospitals prioritized a need in their CHNA. Our results suggest that children's hospitals are more likely to include a similar list of health issues on their CHNAs and that factors beyond county-level health indicators (e.g., organizational mission, regional health indicators, etc.) are operative in hospital decisions to include needs on their CHNAs.

## Introduction

Health services researchers have increasingly focused on the role that health care institutions play in not only providing health care services but also engaging entire communities to improve population health and reduce disparities. One mechanism by which scholars have studied this involvement is in the community benefit activities that nonprofit hospitals, which comprise almost two-thirds of hospitals in the United States, carry out in exchange for tax exemption. Subsequent to the Affordable Care Act (ACA), nonprofit hospitals have been subject to expanded reporting guidelines for their community benefit efforts. As such, there is an opportunity to better understand how general and children's hospitals are assessing needs in their surrounding communities, which likely impacts the development of new population health activities. Hospitals may tailor their community benefit activities to specific populations based on their organizational mission or other local factors. We explore the extent to which children's hospitals are unique in the community health needs they identify as compared with hospitals serving primarily adults. We therefore have an opportunity to assess the content of newly required community benefit reports and how the process of identifying and prioritizing health needs varies across between general and children's hospitals.

Although nonprofit hospitals have been subject to community benefit regulation since the mid-twentieth century, the ACA introduced new reporting requirements to encourage hospitals to focus their community benefit activities on local health needs in the broader community (1). Because more individuals were to be insured with the introduction of the individual mandate and new insurance exchanges and with Medicaid expansion, it was theorized that hospitals might shift some of the benefits they were providing uninsured patients to broader population health activities, which should be documented through the new reporting requirements. As of 2012, all hospitals that are registered as 501(c) (2) organizations must complete a Community Health Needs Assessment (CHNA) every three years and make this information publicly available. Hospitals must follow up this reporting process with a formal implementation plan outlining the subset of identified needs that their organization will address, along with an overview of programmatic goals and strategies (3).

The Internal Revenue Service (IRS), which oversees community benefit reporting, issued guidelines for hospitals to follow in their reporting process. Hospitals are required to identify the needs of their community, prioritize these needs into the most critical or pressing, and identify resources available to address these needs (2). Hospitals may use various methodologies to identify needs but must, at a minimum, consult the following: at least one public health department with knowledge of the community, local residents of the surrounding community, and feedback received on the previous CHNA and/or implementation strategy. The hospital must then undertake and document the process by which they synthesize these data and prioritize a list of the most significant community needs. Despite the guidelines to ensure standardized reporting, there is considerable leeway in the process. Hospitals may give significant weight to primary data, such as using survey or interview methods to include the perspectives of local residents, community leaders, or medical professionals. Hospitals may also rely significantly on secondary data such as county or state-level health indicators (4). Because there is flexibility in the process of identifying and prioritizing local health needs, hospitals within the same community may arrive at different sets of priority health needs. We have no systematic research, however, on the needs being identified by hospitals across the United States. This information is important because hospitals use this process not only to prioritize a set of critical health needs, but to guide population health activities. Whether they vary between children's or general hospitals or reflect county-level health outcomes is important for understanding the population health investments made by hospitals and the extent to which communities will benefit from the engagement of local hospitals around specific health needs.

Previous research on the content of CHNAs and implementation strategies shows that some needs are more commonly identified than others (1, 5–7). For example, social needs and social determinants of health are commonly prioritized as top needs in hospital CHNAs and hospital characteristics were associated with whether these needs were addressed by hospitals in their implementation strategy (8). Clinical needs such as access to care, insurance coverage, and mental health are also commonly identified as community needs, but not all hospitals addressed these necessities in their implementation strategies (9). Other studies have assessed the extent to which identified needs reflect secondary data on health outcomes in the community, and still others have assessed the extent to which community-level factors have shaped the CHNA process. For example, researchers have found that hospitals located in communities with the greatest health needs completed fewer community health activities than hospitals located in areas with lower need (10). Chaiyachati et al. (11) assessed a wide range of sociodemographic characteristics in the community and found that need was unrelated to the amount of spending on community development. Other studies have been conducted at the state level. Pennel et al. (12) analyzed the CHNAs of Texas hospitals and documented wide variation in how CHNAs were reported and in the quality of reports, whereas a content analysis by Beatty et al. (13) examined the degree of collaboration between hospitals and Local Health Departments in Missouri using CHNAs. Both studies described significant variation in the degree of collaboration to produce the CHNA reports. The relatively few studies that have assessed CHNAs after the ACA have not used large national samples or taken into account institutional differences, such as those found between children's and general hospitals in the United States.

Of the more than 6,000 hospitals in the United States, ~230 provide care specifically for patients younger than 18 years of age. These institutions tend to serve large geographic regions (14). As such, their role as a community anchor often differs from general hospitals. Preliminary evidence suggests that children's hospitals dedicate more resources to community benefit efforts than general hospitals (7), but we know very little about the types of activities that children's hospitals undertake and if they are different from other types of hospitals. To date, no systematic investigation has considered the CHNAs of children's hospitals or compared them with general hospitals in the United States.

Although no systematic comparisons exist between children's and general hospitals, the following case studies suggest that children's hospitals may be unique in the community needs that they identify. For example, Nationwide Children's Hospital in Columbus, Ohio, has identified upstream health issues, or the broader social environment that contributes to inequality and chronic disease, to address in their surrounding neighborhood. For example, Nationwide Children's hospital has developed initiatives to improve the quality of available housing, improve employment rates, and elevate neighborhood safety (15, 16). These upstream social determinants of health which the hospital is addressing are associated with a number of chronic diseases including asthma, hypertension, and infant mortality (17–21). The hospital also has implemented an accountable care organization called Partners for Kids, which uses population health strategies financed primarily through Medicaid funding for a much larger population of 330,000 children served by the hospital. This initiative focuses on establishing long-term funding for population health to address community needs identified in their CHNA (22, 23).

Boston Children's Hospital, like many other U.S. general and children's hospitals, was conducting regular CHNAs to guide population health planning before the ACA's new requirements. Based on results from their 2003 CHNA, they developed an innovative asthma initiative that leveraged existing partnerships and networks and included a socioecological approach to addressing child asthma. The activities they've adopted include fostering collaboration with public health departments, providing health education, undertaking case management, and engaging in policy advocacy to reduce health disparities (24). Like Nationwide Children's Hospital, they developed an innovative payment model for high-risk pediatric asthma patients, which has shown a positive return on investment and has allowed them to advocate for health care reform related to pediatric asthma.

A third case study from Children's Hospital Colorado provides data on the ability of Health Impact Assessments (HIAs) to be used as part of the CHNAs to make effective recommendations for programs and policy changes that would have a positive impact on population health outcomes. Because hospitals are asked to develop strategies to address identified needs, this approach may help hospitals meet the broader community benefit goals of addressing the most critical local issues related to community health. At Children's Hospital Colorado, they conducted pilot case studies using HIAs to inform the community benefit decision-making process. The case studies included specific needs related to children's health: the relationship between parental marijuana use and child abuse or neglect and on adolescent behavioral health. A third case study focused on a specific community served by the hospital. Preliminary findings from the use of HIAs suggest that this approach helped to synthesize feedback from multiple partners and strengthen ties between stakeholders in the process of determining the priority list of needs. The case studies also suggested that this process was effective at identifying evidence-based strategies to address community health needs (25). This approach may be an effective way to move hospital community benefit activities upstream through the use of evidence-based programs and policy advocacy.

The available case studies suggest that children's hospitals are undertaking novel approaches to population health that transcend the traditional focus on charity care and patient engagement in community benefit investments. These case studies suggest that children's hospitals are undertaking approaches aimed at general population health improvement and the reduction of health disparities as part of their community benefit process. In other words, hospitals are responding to the social and non-medical needs identified in their CHNAs with new strategies to elevate local health outcomes above and beyond the acute medical care they provide. The goal of the present study is to assess whether children's hospitals are unique in their approach to identifying community needs and which community and organizational characteristics are associated with decisions to prioritize needs in their federally mandated CHNAs.

## Methods

### Sample

To build a data set of hospital community benefit practices, we brought together several types of data, including hospitals' prioritized needs from their CHNAs; organizational characteristics; and variables related to the county, state, and region. Data on hospitals' needs were gathered from the publicly available CHNAs of all nonprofit children's hospital members of the Children's Hospital Association (*n* = 234) and a 20% random sample of all nonprofit general, nonspecialty hospitals in the United States generated from the American Hospital Association Annual Survey (*n* = 617) (26). We combined these two samples for inclusion in the data set, which represented 851 hospitals. We downloaded each hospital's CHNA and implementation strategy. If these documents were not publicly available on the organization's website, we e-mailed and/or called the contact listed to request a copy of the report. If we were unable to make contact with the hospital, they were dropped from the sample. After dropping hospitals with missing information, the total sample was 737. All CHNAs and implementation strategies were collected and coded in 2018 and 2019. Because hospitals complete the reporting process every 3 years, and had the option of starting in either 2012 or 2013, the CHNAs and implementation strategies included in our data set range from 2015 to 2018. Data on organizational characteristics came from the 2015 American Hospital Association Annual Survey and 2015 Children's Hospital Association Population Health Survey. To assess community characteristics, we included the county in which each hospital is located. Data on county health characteristics came from the 2015 County Health Rankings database and the Centers for Disease Control and Prevention's National Vital Statistics System (27, 28). Additional community and county characteristics were sourced from the Area Health Resource File with data from 2015 (29).

### Coding

The authors and a research assistant reviewed and coded each CHNA for the hospital's list of prioritized health needs and the corresponding implementation plan for whether a prioritized need was addressed with a specific intervention strategy. Hospitals are not required to address each need that they prioritize, but they must provide an explanation if they do not address a prioritized need. For most CHNAs, prioritized needs are clearly listed along with the process described for selecting these top needs. In a few situations, the CHNAs did not contain a clear list of health needs. In these cases, we met as a research team to review the CHNAs and collaboratively code them. In addition, we selected a number of CHNAs to code independently to ensure reliability. At several points during the coding process, we coded the same files independently to ensure reliability. Our end result was a list of the top five health needs identified by each hospital.

After coding the top needs for all hospitals, we determined the five most commonly identified health needs across the adults' and children's samples, and for each of these five health needs (see Table 1) we created dichotomous variables for whether each condition was ranked in the top five on a hospital's CHNA. We chose to take a 20% random sample of nonprofit general hospitals, rather than assess all general nonprofit hospitals, because the process of retrieving and coding CHNAs for this number of hospitals was very labor-intensive. To confirm that our analytic sample was representative of the population, we compared the organizational characteristics of the 737 hospitals within our sample to those of the 2,779 nonprofit hospitals captured in the 2015 American Hospital Association Annual Survey. We found that our sample was highly comparable to the general nonprofit hospital population across a range of characteristics (bed size, system membership, rural or urban location, critical access status, and academic medical center designation).

**Table 1:** Descriptive statistics for analytic sample by children's and general hospitals.

	**Children's hospitals**		**General hospitals**	
	***N***	**%**	***N***	**%**
**Hospitals**	175	23.74	562	76.26
**Hospital community health need assessment items**				
CHNA: access	113	64.57	236	41.99
CHNA: chronic illness	136	77.71	329	58.54
CHNA: mental health	137	78.29	281	50.00
CHNA: substance use	60	34.29	153	27.22
CHNA: social needs	75	42.86	128	22.78
**Key county characteristics**	**Mean**	**SD**	**Mean**	**SD**
Primary care providers per population	1,129	334		
Age adjusted premature death	336	73	340.66	84.21
Poor mental health ms	3.41	0.51	3.32	0.97
Drug overdose rate	17.24	5.7	15.59	6.24
Severe housing problem	20.69	5.24	16.96	5.88
**Hospital characteristics**	***N***	**%**	***N***	**%**
Bed size: fewer than 50	14	8.00	172	30.60
Bed size: 50–199	84	48.00	194	34.52
Bed size: 200–399	37	21.14	119	21.17
Bed size: >400	40	22.86	77	13.70
Hospital system member	132	75.43	405	72.06
**Community characteristics**	***N***	**%**	***N***	**%**
State expanded Medicaid	107	61.14		
County rural	2	1.14	199	35.41
Region: Northeast	35	20.00	107	19.04
Region: Midwest	45	25.71	200	35.59
Region: South	59	33.71	144	25.62
Region: West	36	20.57	111	19.75
	**Mean**	**SD**	**Mean**	**SD**
Non-Hispanic white population	65.93	15.99	79.25	17.21
Median age	35.9	2.98	3.9	4.5

*N = 737*.

### Measures

In this study, our dependent variable is whether a hospital identified one of the five most common needs in its top five prioritized needs. We ran a separate model for each of the most commonly identified needs across the sample: mental health, access to care, chronic illness, substance misuse, and social needs. Our focal independent variable is whether the hospital is a children's hospital. We also controlled for county-level health outcome characteristics that were directly related to the identified needs considered within the study in order to assess the extent to which hospitals rank needs that are evidence in available secondary health data at the county level. We chose to use county-level data because hospitals often use counties as their service area and in their definitions of the surrounding community (4). After reviewing the available county-level measures, we selected one overlapping health variable to pair with each need. Although in some cases there were multiple needs that were related, we ran *t*-tests to determine if variables had a relationship with the identification of a need in hospitals' CHNAs and then met as a team to determine the variables that fit based on their conceptual and/or statistical compatibility. See Table 2 for the county-level variables that were paired with each CHNA-identified need. In addition, we controlled for county-level demographic characteristics, including whether hospitals were located in a rural county (compared with located in metro counties) based on data from the Area Health Resource File and the rural–urban continuum codes from the U.S. Department of Agriculture. Median age in a county, percentage of white residents in a county, whether the state had expanded Medicaid access after the ACA, and the broader region of the hospital served as control variables as well. We selected this set of control variables based on prior conceptual work suggesting a relationship between community-level demographics and institutional investments (30). In addition, we build on previous studies of community benefit which suggest that broader county and state environments shape hospital decision making (31).

**Table 2:** Logistic regression health need recognition by hospital and community characteristics.

	**Odds ratio (SD)**	**[95% Confidence interval]**	**Odds ratio (SD)**	**[95% Confidence interval]**	**Odds ratio (SD)**	**[95% Confidence interval]**
**Mental health**						
Children's hospital (Ref: General hospitals)	3.63 (0.73)^***^	2.44–5.40	3.61 (0.75)^***^	(2.40–5.42)	4.31 (0.97)^***^	2.78–6.69
County poor mental health days	0.93 (0.08)	0.79–1.10	0.92 (0.08)	(0.77–1.09)	0.95 (0.08)	0.80–1.13
Bed size 1 (Ref: >400)	–	–	1.14 (0.29)	(0.70–1.87)	0.85 (0.24)	0.48–1.49
Bed size 2 (Ref: >400)	–	–	1.35 (0.32)	(0.85–2.13)	1.14 (0.28)	0.70–1.84
Bed size 3 (Ref: >400)	–	–	1.09 (0.28)	(0.66–1.79)	1.01 (0.27)	0.60–1.69
System membership	–	–	1.41 (0.24)^*^	(1.00–1.97)	1.45 (0.26)^*^	1.02–2.06
State Medicaid expansion	–	–	–	–	0.84 (0.19)	0.55–1.30
Population white	–	–	–	–	1.13 (0.07)^*^	1.00–1.28
Median age	–	–	–	–	1.00 (0.02)	0.96–1.05
County rural	–	–	–	–	0.96 (0.22)	0.62–1.50
Region: Northeast (Ref: Midwest)	–	–	–	–	0.92 (0.22)	0.58–1.47
Region: South (Ref: Midwest)	–	–	–	–	0.60 (0.15)^*^	0.37–0.97
Region: West (Ref: Midwest)	–	–	–	–	1.48 (0.36)	0.92–2.38
**Access**						
Children's hospital (Ref: General hospitals)	2.63 (0.49)^***^	1.83–3.78	2.65 (0.50)^***^	1.84–3.84	2.56 (0.52)^***^	1.72–3.82
Primary care provider rate per 1,000 county residents	1.00 (0.00)	1.00–1.00	1.00 (0.00)	1.00–1.00	1.00 (0.001)	1.00–1.00
Bed size 1 (Ref: >400)	–	–	1.13 (0.29)	0.68–1.85	1.18 (0.34)	0.68–2.07
Bed size 2 (Ref: >400)	–	–	1.11 (0.25)	0.71–1.73	1.16 (0.28)	0.73–1.86
Bed size 3 (Ref: >400)	–	–	1.17 (0.30)	0.72–1.92	1.18 (0.31)	0.71–1.96
System membership	–	–	1.20 (0.21)	0.86–1.68	1.11 (0.20)	0.78–1.58
State Medicaid expansion	–	–	–	–	1.04 (0.23)	0.69–1.60
Population white	–	–	–	–	0.87 (0.05)^*^	0.78–0.98
Median age	–	–	–	–	1.02 (0.02)	0.97–1.07
County rural	–	–	–	–	1.22 (0.28)	0.78–1.92
Region: Northeast (Ref: Midwest)	–	–	–	–	0.62 (0.15)^*^	0.39–0.99
Region: South (Ref: Midwest)	–	–	–	–	1.35 (0.32)	0.85–2.16
Region: West (Ref: Midwest)	–	–	–	–	1.83 (0.43)	1.16–2.90
**Substance use**						
Children's hospital (Ref: General hospitals)	1.34 (0.25)	0.93–1.93	1.42 (0.28)	0.97–2.08	1.79 (0.40)^**^	1.16–2.76
County drug death rate	1.03 (0.01)^*^	1.00–1.06	1.03 (0.01)^*^	1.00–1.06	1.04 (0.02)^**^	1.01–1.07
Bed size 1 (Ref: >400)	–	–	1.47 (0.40)	0.86–2.50	1.28 (0.40)	0.70–2.35
Bed size 2 (Ref: >400)	–	–	1.43 (0.36)	0.88–2.33	1.2 (0.32)	0.73–2.06
Bed size 3 (Ref: >400)	–	–	0.64 (0.19)	0.36–1.15	0.59 (0.18)	0.32–1.08
System membership	–	–	0.73 (0.13)	0.51–1.05	0.78 (0.15)	0.53–1.13
State Medicaid expansion	–	–	–	–	0.92 (0.22)	0.58–1.46
Population white	–	–	–	–	1.36 (0.10)^***^	1.17–1.57
Median age	–	–	–	–	1.02 (0.03)	0.96–1.07
County rural	–	–	–	–	0.53 (0.13)^*^	0.32–0.86
Region: Northeast (Ref: Midwest)	–	–	–	–	1.78 (0.44)^*^	1.09–2.88
Region: South (Ref: Midwest)	–	–	–	–	0.99 (0.27)	0.58–1.68
Region: West (Ref: Midwest)	–	–	–	–	0.66 (0.19)	0.38–1.15
**Chronic illness**						
Children's hospital (Ref: General hospitals)	2.49 (0.50)^***^	1.68–3.69	2.20 (0.45)^***^	1.47–3.30	1.89 (0.41)^**^	1.23–2.91
Age adjusted premature death	1.00 (0.001)	1.00–1.00	1.00 (0.001)	1.00–1.00	1.00 (0.001)	1.00–1.00
Bed size 1 (Ref: >400)	–	–	0.60 (0.15)^*^	0.37–0.99	0.85 (0.24)	0.48–1.49
Bed size 2 (Ref: >400)	–	–	1.15 (0.28)	0.72–1.84	1.36 (0.34)	0.83–2.23
Bed size 3 (Ref: >400)	–	–	0.92 (0.24)	0.55–1.53	0.96 (0.25)	0.57–1.62
System membership	–	–	0.85 (0.15)	0.60–1.20	0.85 (0.16)	0.59–1.23
State Medicaid expansion	–	–	–	–	0.82 (0.18)	0.53–1.27
Population white	–	–	–	–	0.96 (0.06)	0.85–1.09
Median age	–	–	–	–	0.99 (0.02)	0.94–1.04
County rural	–	–	–	–	0.69 (0.16)	0.44–1.08
Region: Northeast (Ref: Midwest)	–	–	–	–	1.81 (0.46)^*^	1.10–2.96
Region: South (Ref: Midwest)	–	–	–	–	0.95 (0.25)	0.57–1.58
Region: West (Ref: Midwest)	–	–	–	–	0.83 (0.20)	0.52–1.33
**Social needs**						
Children's hospital (Ref: General hospitals)	2.26 (0.43)^***^	1.56–3.28	2.27 (0.44)^***^	1.56–3.33	2.39 (0.50)^***^	1.59–3.59
County percent with severe housing issues	1.03 (0.01)^*^	1.01–1.06	1.03 (0.02)^*^	1.00–1.06	1.05 (0.02)^*^	1.00–1.09
Bed size 1 (Ref: >400)	–	–	0.82 (0.23)	0.47–1.42	0.73 (0.23)	0.40–1.35
Bed size 2 (Ref: >400)	–	–	0.79 (0.19)	0.49–1.28	0.74 (0.19)	0.45–1.22
Bed size 3 (Ref: >400)	–	–	1.00 (0.27)	0.59–1.69	0.90 (0.25)	0.53–1.55
System membership	–	–	0.82 (0.16)	0.57–1.20	0.77 (0.15)	0.53–1.13
State Medicaid expansion	–	–	–	–	1.45 (0.36)	0.89–2.36
Population white	–	–	–	–	0.99 (0.08)	0.85–1.16
Median age	–	–	–	–	1.06^*^ (0.03)	1.00–1.11
County rural	–	–	–	–	0.76 (0.21)	0.45–1.30
Region: Northeast (Ref: Midwest)	–	–	–	–	0.40 (0.11)^**^	0.23–0.70
Region: South (Ref: Midwest)	–	–	–	–	0.97 (0.26)	0.57–1.65
Region: West (Ref: Midwest)	–	–	–	–	0.88 (0.24)	0.52–1.50

*Estimate [95% Confidence interval]*.

* *p < 0.05*;

** *p < 0.01*;

*** *p < 0.001*.

*N = 737*.

### Analytic Strategy

To assess the relationship between hospital characteristics; county, state, and regional factors; and decisions to include needs in a hospital CHNA, we employed descriptive statistics to understand the percentage of hospitals identifying each need and then performed multivariate logistic regression to understand the impact of multiple factors on hospital decisions to identify each need. We constructed five models for each of the most common health needs and considered the odds of identifying each need, based on a variety of hospital and community-level characteristics. In each model, we assessed the relationship between identifying each need, whether an organization was a children's hospital as compared to being a general hospital, an overlapping county health outcome, and both hospital and community characteristics. All statistical analyses were conducted using Stata 16.

## Results

Descriptive analysis shows that 24% of the hospitals in our sample were children's hospitals. Children's hospitals were more likely to identify each of the top five most common community needs as compared with non-children's hospitals.

**Figure 1:**
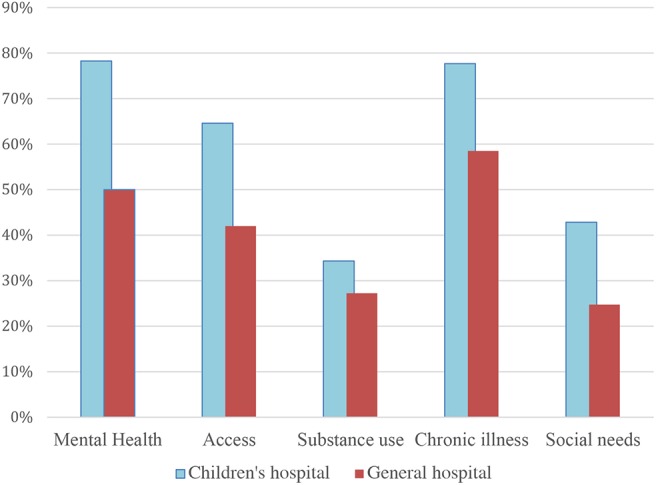
Percent of hospitals identifying need on community health need assessment by children's and general hospitals. *N* = 737 hospitals.

The gaps were largest for access and mental health (see Figure 1). For example, 65% of children's hospitals identified access compared with 42% of general hospitals. For chronic illness, 78% of children's hospitals identified this need vs. 59% of general hospitals. We found that 78% of children's hospitals identified mental health compared with 50% of general hospitals. Substance misuse was identified in the CHNAs of 34% of children's hospitals and 27% of general hospitals. Finally, 43% of children's hospital identified social needs compared with 25% of general hospitals.

Mental health is the need most prioritized on implementation plans by children's hospitals (68% of those who identified the need), whereas chronic illness is that need for general hospitals (53% of those identifying the need) (Table 3). All five needs were more likely to be represented on the implementation strategies of children's hospitals. If a need was prioritized on a CHNA, general hospitals were slightly more likely to have adopted one or more programs to address all needs—with the exception of mental health—in their implementation strategy. For both children's hospitals and general hospitals, social needs were the least likely to be addressed on the implementations strategy, despite being prioritized on the CHNA.

**Table 3:** Hospital need recognition on community health need assessment and prioritization on implementation Plan comparing children's to general hospitals.

	**Mental health**	**Access**	**Substance use**	**Chronic illness**	**Social needs**
**Need reported on community health needs assessment (CHNA)**					
Children's hospital	78.29%	64.57%	34.29%	77.71%	42.86%
General hospital	50.00%	41.99%	27.22%	58.54%	22.78%
**Need included on implementation plan**					
Children's hospital	68.15%	54.14%	27.39%	65.61%	24.84%
General hospital	41.85%	35.95%	22.40%	52.95%	14.73%

*N = 737*.

In terms of institutional characteristics across the sample, children's hospitals in our sample are more likely to be in the largest category of bed size: 23% of children's hospitals have 400 or more beds, whereas only 14% of general hospitals are in this category. Not surprising, children's hospitals are less likely to be in rural areas (only 1% compared with 35% of general hospitals). Key county-level characteristics are similar between the full sample and the children's hospital subsample (Table 1).

Our multivariate results provide a fuller understanding of how organizational, county, state, and regional factors relate to the identification of community health needs. Our results show that for the top five most common conditions across CHNAs in our sample, children's hospitals are more likely than general hospitals to identify each of these needs. Only two of the paired county health variables were significant, suggesting that hospitals are likely considering additional factors beyond whether secondary data indicate a critical health concern. For substance misuse, hospitals were more likely to identify this need if the county-level overdose rate was higher. Hospitals also had higher odds of including substance misuse as a top need if they were located in the Northeast, compared with the Midwest, or in counties where there was a greater proportion of white residents. Hospitals in rural counties were also less likely to identify substance misuse as a top need.

The county health ranking paired with social needs was also significant, suggesting that hospitals were more likely to identify social needs as a priority when there were more severe housing problems in the county. Hospitals also had higher odds of identifying social needs when the median county age was higher. Hospitals in the Northeast were less likely to identify social needs compared with hospitals in the Midwest. For access to care, hospitals are less likely to identify this need in the Northeast, compared with the Midwest, and when there are more white residents in the county. For chronic illness, hospitals are more likely to identify this need in the Northeast. In terms of identifying access to mental health, hospitals in systems and hospitals located in counties with a greater proportion of white residents are more likely to have mental health as a top need on their CHNA. Hospitals located in the South are less likely to identify mental health compared with hospitals in the Midwest. Finally, for social needs, children's hospital is significant and positive, as is severe housing problem, median age, and Northeast region.

## Discussion

Our findings provide important insight into the decisions that hospitals make to include health needs as top priorities in their CHNAs. A past study found that hospitals that ranked needs at the top of their list of prioritized needs are more likely to have accompanying strategies to address these needs in their implementation strategies (32). As such, hospital decisions to prioritize a need in their CHNA are likely related to the presence of actionable strategies in the corresponding implementation strategy. When looking at the top five needs identified in all sample hospital CHNAs (access to care, chronic illness, substance misuse, mental health, and social needs), we find that children's hospitals have greater odds of including these particular needs at the top of their prioritized needs but that other organizational characteristics are not predictive of which needs the hospitals prioritize. These findings suggest that primarily adult-serving hospitals likely have greater variation in the needs they identify and therefore are less likely to include each of the most common needs in their prioritized list. Children's hospitals may simply be more similar to one another, given their unique mission and patient population.

For example, the most common conditions among admitted patients varies greatly between children and adults. While the top five conditions for children include respiratory conditions (pneumonia, asthma, bronchitis), mood disorders, and appendicitis, the top five conditions among hospitalized adults are mental illness (mood disorders, schizophrenia), skin infections, diabetes, and biliary tract disease (33). Although substance abuse is the number seven cause of hospitalization among adults, it does not appear in the top 10 for children. Mental health conditions appear in the top five conditions for both adults and children, but represent the top two conditions for adults and are only the 4th most common condition among children.

Children are most likely to be hospitalized at general hospitals (including children's hospitals within general hospitals), but when they are hospitalized at free-standing children's hospitals they are more likely to be neonates, have higher condition severity, and have longer lengths of stay (34). Admissions to freestanding children's hospitals are also more likely to be for children with medical complexity. In terms of patient populations, children's hospitals serve larger geographic regions (35) than their general hospital counterparts and therefore may see a subset of needs as essential overall to improving population health regardless of varying public health needs in their surrounding communities. General hospitals, by contrast, may be more willing to identify needs specific to their surrounding community.

To better understand the factors that hospitals may consider when deciding whether to include a health need in their prioritized list of top five health concerns, we included a number of variables in our multivariate models. For each of the most common needs, we paired a corresponding county health indicator to assess whether hospitals relied heavily on secondary health data when ranking top needs. In most cases, these county variables did not seem to influence hospital decisions. Two exceptions included substance misuse and social needs. We interpret these findings to mean that many hospitals, and children's hospitals in particular, may be including health needs that are broader than the immediate county or reflect other priorities, such as needs that are considered highly relevant by local residents or by the hospital. One previous study found a high level of overlap between county health indicators and the prioritization of needs in CHNAs among general hospitals (32). With children's hospitals included, our findings may reflect a broader focus on regional, rather than county-level, health needs. It is also possible that both general and children's hospitals may identify needs that are compatible with their mission or expertise rather than solely relying on available public health data for the surrounding county.

Other county-level and regional characteristics were significant and provide additional insight into how hospitals make decisions about which needs to prioritize in their CHNAs. The fact that hospitals were more likely to identify mental health and substance misuse if the county had a higher proportion of white residents suggests that the demographic profiles of a community may shape decision-making. Given that mental health and substance misuse are commonly identified non-medical needs that require expanded hospital expertise and community-based partnerships, hospitals may be less likely to acknowledge these needs in racially diverse populations. It is possible that implicit bias, a phenomenon well documented in medical institutions (36, 37), may impact decision-making at the organizational level or that this is an example of the institutionalized prejudices still prevalent in society. In a similar vein, we find that hospitals are less likely to identify substance misuse if they are in rural counties, suggesting that hospitals may be aware of resources that are limited in rural areas (38) when they make decisions to rank a need as a top priority in their CHNA.

Finally, we find strong evidence that needs vary across regions. Some of these differences may reflect the prevalence of health needs in a region, such as hospitals having higher odds of identifying substance misuse in the Northeast, where rates of opioid addiction are currently higher than in many other regions. Mental health is less likely to be identified in the South, however, which suggests that broader cultural values and stigma may be associated with decisions to identify certain health conditions as top community needs. Future research should assess the extent to which social, demographic, and cultural factors shape decisions to identify specific health needs as priorities on hospitals' CHNAs.

### Limitations

Our findings provide insight into community benefit decision-making for a large national sample of hospitals, but they are limited for several reasons. We rely on self-reported data from hospitals to assess what needs they rank as the most critical in their communities. Hospitals come to these conclusions in a variety of ways, including through collecting community input and establishing a ranking process for prioritizing needs in the final CHNA. Based on our use of available secondary data, we are unable to assess reasons for prioritizing a need in the CHNA. Future research should include the use of qualitative methods to better understand decision making processes and opportunities to align hospital community benefit investments with local need. Although hospitals are required to make public their most recent CHNA, a number of hospitals did not post these documents publicly or respond to our requests for a copy and therefore had to be excluded from the sample. Our study focuses primarily on the prioritizing of top health needs within hospital CHNAs and does not analyze the odds of addressing prioritized needs in the hospital's corresponding implementation strategy. We do report results on the rate by which each need is addressed with new programs in the implementation strategy, but future studies should consider which factors increase the likelihood that hospitals develop actionable and evidence-based strategies to address the needs that they identify in their CHNAs and if these differ by type of hospital. Additionally, we rely on county-level data to assess the broader community in which hospitals operate. Counties are an imperfect proxy for communities and it's likely that significant variation in health outcomes exists within counties, Nonetheless, using counties allowed us to include the most consistent source of local health data to include the health profile of counties where hospitals operate and are making community benefit investment decisions.

### Public Health Implications

Because most hospitals in the United States are 501(c) (2) tax-exempt organizations, new community benefit requirements associated with the ACA aimed to increase accountability through new reporting guidelines that require hospitals to consider community health needs when making decisions about community benefit investments. Given the significant amount of flexibility that the IRS provides in completing CHNAs, our study provides insight into the factors associated with the designation of specific issues as priority health needs and how different types of hospitals may approach this task. For hospitals to contribute to population health improvement in their surrounding communities, policymakers should provide additional guidance, as well as relevant incentives to encourage hospitals to prioritize critical local health needs in their CHNAs and identify evidence-based programs to address prioritized needs.

## Data Availability Statement

The datasets generated for this study are available on request to the corresponding author.

## Author Contributions

BF and CC contributed to the conception and design of the study and read and approved the submitted version. CC organized the data, performed the statistical analysis, contributed to manuscript writing, and edited the manuscript. BF wrote the first draft of the manuscript.

### Conflict of Interest

The authors declare that the research was conducted in the absence of any commercial or financial relationships that could be construed as a potential conflict of interest.
